# Prognostic Role of Specific *KRAS* Mutations Detected in Aspiration and Liquid Biopsies from Patients with Pancreatic Cancer

**DOI:** 10.3390/genes15101302

**Published:** 2024-10-07

**Authors:** Tereza Hálková, Bohuš Bunganič, Eva Traboulsi, Marek Minárik, Miroslav Zavoral, Lucie Benešová

**Affiliations:** 1 Centre for Applied Genomics of Solid Tumors (CEGES), Genomac Research Institute, Drnovská 1112/60, 161 00 Prague, Czech Republic; 2 Department of Biochemistry, Faculty of Science, Charles University, Hlavova 8/2030, 128 00 Prague, Czech Republic; 3 Department of Medicine, First Faculty of Medicine, Charles University and Military University Hospital, U Vojenské Nemocnice 1200, 169 02 Prague, Czech Republic; 4 Department of Pathology, Military University Hospital Prague, U Vojenské Nemocnice 1200, 169 02 Prague, Czech Republic; 5 Department of Analytical Chemistry, Faculty of Science, Charles University, Hlavova 8/2030, 128 00 Prague, Czech Republic; mminarik@email.com

**Keywords:** *KRAS*, mutation type, prognosis, EUS-FNB, ctDNA, liquid biopsy, pancreatic cancer

## Abstract

**Background/Objectives:** Although the overall survival prognosis of patients in advanced stages of pancreatic ductal adenocarcinoma (PDAC) is poor, typically ranging from days to months from diagnosis, there are rare cases of patients remaining in therapy for longer periods of time. Early estimations of survival prognosis would allow rational decisions on complex therapy interventions, including radical surgery and robust systemic therapy regimens. Understandably, there is great interest in finding prognostic markers that can be used for patient stratification. We determined the role of various *KRAS* mutations in the prognosis of PDAC patients using biopsy samples and circulating tumor DNA. **Methods:** A total of 118 patients with PDAC, clinically confirmed by endoscopic ultrasound-guided fine-needle aspiration biopsy (EUS-FNB), were included in the study. DNA was extracted from cytological slides following a standard cytology evaluation to ensure adequacy (viability and quantity) and to mark the tumor cell fraction. Circulating tumor DNA (ctDNA) was extracted from plasma samples of 45 patients in stage IV of the disease. *KRAS* mutations in exons 12 and 13 were detected by denaturing capillary electrophoresis (DCE), revealing a minute presence of mutation-specific heteroduplexes. Kaplan–Meier survival curves were calculated for individual *KRAS* mutation types. **Results:** *KRAS* mutations were detected in 90% of tissue (106/118) and 44% of plasma (20/45) samples. All mutations were localized at exon 2, codon 12, with G12D (GGT > GAT) being the most frequent at 44% (47/106) and 65% (13/20), followed by other types including G12V (GGT > GTT) at 31% (33/106) and 10% (2/20), G12R (GGT > CGT) at 17% (18/106) and 10% (2/20), G12C (GGT/TGT) at 5% (5/106) and 0% (0/20) and G12S (GGT/AGT) at 1% (1/106) and 5% (1/20) in tissue and plasma samples, respectively. Two patients had two mutations simultaneously (G12V + G12S and G12D + G12S) in both types of samples (2%, 2/106 and 10%, 2/20 in tissue and plasma samples, respectively). The median survival of patients with the G12D mutation in tissues was less than half that of other patients (median survival 101 days, 95% CI: 80–600 vs. 228 days, 95% CI: 184–602), with a statistically significant overall difference in survival (*p* = 0.0080, log-rank test), and furthermore it was less than that of all combined patients with other mutation types (101 days, 95% CI: 80–600 vs. 210 days, 95% CI: 161–602, *p* = 0.0166). For plasma samples, the survival of patients with this mutation was six times shorter than that of patients without the G12D mutation (27 days, 95% CI: 8–334 vs. 161 days, 95% CI: 107–536, *p* = 0.0200). In contrast, patients with detected *KRAS* G12R in the tissue survived nearly twice as long as other patients in the aggregate (286 days, 95% CI: 70–602 vs. 162 days, 95% CI: 122–600, *p* = 0.0374) or patients with other *KRAS* mutations (286 days, 95% CI: 70–602 vs. 137 days, 95% CI: 107–600, *p* = 0.0257). **Conclusions:** Differentiation of specific *KRAS* mutations in EUS-FNB and ctDNA (above all, the crucial G12D and G12R) is feasible in routine management of PDAC patients and imperative for assessment of prognosis.

## 1. Introduction

Pancreatic carcinoma, particularly the most common subtype, pancreatic ductal adenocarcinoma (PDAC), is one of the most fatal malignancies. It is the 12th most common cancer worldwide, with the highest incidence and mortality rates in advanced countries [1]. The insidious nature of PDAC consists predominantly of the absence or nonspecificity of its symptoms, resulting in late diagnosis, usually in an inoperable stage of the disease. Most patients are diagnosed with metastatic disease (stage IV) [2], when the disease progression is very rapid, with survival time typically being less than 5 months from diagnosis. Only in a minority of cases is the disease diagnosed when the tumor is surgically resectable, which, at present provides the only chance for a cure. Even if the tumor is removed completely and has not spread to any lymph nodes, the five-year survival does not exceed 40% [3].

As PDAC patients often exhibit a poor performance status and the effect of treatment is negligible, systemic therapy should be carefully considered with regard to the quality of life, especially for unresectable tumors. On the other hand, individual cases of patients with significantly longer overall survival do exist (based on our experience, about 2% of patients in stage IV survive for more than 1.5 years), and these patients benefit from surgical or systemic therapy. However, no reliable prognostic biomarkers are currently available to estimate the prognosis of patients with PDAC, and their discovery is therefore highly desirable.

From a genetic point of view, the carcinogenesis of PDAC is usually triggered by a mutation in the *KRAS* gene, which is an early and characteristic genetic event in PDAC [4]. *KRAS* is a proto-oncogene encoding a small G-protein, Kras, which is localized in the inner surface of the plasma membrane and transmits signals regulating cell growth, proliferation and survival, from the cell surface receptor into the nucleus [5]. Ras proteins cycle between GTP-bound active and GDP-bound inactive states. Ras activation (exchange of GDP for GTP) is initiated by guanine exchange factors (GEFs). Depending on the specific GEF protein, activated Ras initiates RAS/RAF/MAPK, RAS/PI3K/PDK1/AKT or RAS/RAL signal transduction pathway. Under physiological conditions, Ras is again inactivated in a short time by the hydrolysis of GTP [5,6,7,8]. However, *KRAS* mutations make the hydrolysis of GTP-bound Ras impossible, leading to permanent activity of the Ras oncoprotein, which subsequently interacts with various effectors. Thus, its role in affected cells becomes much more complex than the role of Ras protein in healthy cells [9].

Due to its frequent occurrence in PDAC tissue samples (about 90%), the prognostic value of *KRAS* mutation status has traditionally been discussed. In previous studies, we presented routine assessments of *KRAS* mutations in cytological smears of bioptic PDAC samples [10,11,12], and, in agreement with investigators from other groups [13,14,15,16,17], we have not observed any correlation between the presence or absence of *KRAS* mutation and survival [11]. There is one older meta-analysis of 17 *KRAS* studies in PDAC [18] whose results report significantly shorter overall survival (OS) for patients harboring *KRAS* mutations compared to those without them. Although this work has certain limitations, in particular the low number of patients (10 studies included fewer than 80 patients) and the low detection of *KRAS* mutations (11 studies reported less than 70% detection of *KRAS* mutations), it still contains several important works suggesting that *KRAS* mutations certainly play some role in the prognosis of PDAC patients, but the question is to what extent they are involved and how.

For example, as shown in a study performed in non–small cell lung cancer (NSCLC) cell lines, not all activating *KRAS* mutations are clinically equivalent because various forms of Ras oncoproteins exhibit different types of biological behavior, have different effects on downstream signaling pathways and thus may also have different effects on patient prognosis [19]. More specifically, Kras G12D activates PI3K and MAPK signaling, whereas the presence of Kras G12C or Kras G12V activates RAL signaling and reduces growth-factor-dependent AKT activation. Depending on the specific *KRAS* mutation in the tumor tissue, differences in progression free survival (PFS) were observed in the same study. NSCLC patients with *KRAS* G12C and G12V had worse PFS than patients with other *KRAS* mutations or wild-type *KRAS* [19]. However, as demonstrated in a study by Eser et al., activation of downstream signaling molecules is not only allele-specific but also tissue-specific. It has been shown that the Pdk1 effector from the PI3K pathway is essential for *KRAS* G12D-driven PDAC but not for G12D-driven lung cancer [20]. It is therefore possible that the role of individual *KRAS* mutations in downstream signaling may differ between NSCLC and PDAC. Nevertheless, emerging scientific work supports the assumption of different impacts of specific *KRAS* mutations on prognosis even in PDAC patients [15,21,22].

In addition to tissue samples, circulating tumor DNA (ctDNA) is often studied in PDAC patients. It is short, fragmented DNA released from the tumor mass into the bloodstream of cancer patients. Due to its exclusive origin in cancerous cells, ctDNA harbors cancer-specific aberrations, such as somatic mutations [23]. Therefore, the presence of a *KRAS* mutation in the plasma indicates the presence of ctDNA. As ctDNA-positive patients with various types of solid tumors, including PDAC, have worse prognosis than ctDNA-negative patients [24,25], the presence of a *KRAS* mutation in ctDNA is also usually related to a worse prognosis.

The incidence of ctDNA in plasma correlates with the stage of the disease, and typically, it is most common in patients with metastatic disease (stage IV) [26]. Undoubtedly, the most important advantage of ctDNA testing is non-invasive sampling, allowing for *KRAS* testing without a biopsy-associated burden on the patient. Another advantage is that a single plasma sample contains all the mutated alleles present in the tumor tissue. This does not apply to endoscopic ultrasound-guided fine-needle aspiration biopsy (EUS-FNB) samples, in which the collected tissue is only a minimal part of the tumor tissue and is, moreover, inhomogeneous and pervaded with fibers, giving a highly heterogeneous representation of the mutated cells. Although ctDNA testing is not as sensitive as the determination of *KRAS* mutations from a tissue sample, it may be a useful tool in PDAC cases where the tissue biopsy is contraindicated or when repeated sampling is required.

In the current work, we therefore verified the prognostic role of individual *KRAS* mutations in our cohort of 118 PDAC patients and assessed whether individual *KRAS* mutations could serve as useful prognostic markers for managing disease and guiding rational treatment decisions that would ensure the longest survival with an adequate quality of life. In addition, we introduced in a subgroup of 45 patients a simple and low-cost method for *KRAS* mutation detection from ctDNA as a source material, including the determination of mutation subtypes.

## 2. Materials and Methods

### 2.1. Patients and Samples

A total of 118 patients aged 67.9 ± 9.4 years were prospectively enrolled in the study. Diagnosis of PDAC was based on EUS with an FNB collection and subsequent cytological assessment of the aspirate.

A tissue biopsy sample was obtained from each of the 118 patients at the time of diagnosis, and additionally, a blood sample for ctDNA analysis was collected from every patient in stage IV of the disease who agreed to the collection (45/58) at the time of diagnosis before initiation of any treatment. All patients signed the study’s informed consent form, and the study protocol was approved by the ethics committee of the Military University Hospital in Prague.

EUS was performed using a GF UCT 180 linear echoendoscope (Olympus, Tokyo, Japan) and a sonographic system (Aloka Pro Sound Alpha 10, Tokyo, Japan). Tumor FNBs were collected using standard 22-gauge needles (EZ Shot 2, Olympus, Tokyo, Japan) and Expect needles (Boston Scientific, Natick, MA, USA), intended for standard cytological assessments. The cellular material was spread on cytological slides on-site, and dry cytological smears were examined by the pathologist after being stained using the May–Grünwald–Giemsa technique. Furthermore, the pathologist highlighted areas with a major incidence of tumor cells (approx. 80%) on the slides.

Plasma was separated by centrifugation from the blood samples immediately after collection and was then frozen to −18 °C.

### 2.2. Nucleic Acid Extraction and Detection of KRAS Mutations

Tumor cells were dissected from the cytological preparations from areas with major incidences of tumor cells and transferred to a microtube. DNA was extracted using a Recover All Total Nucleic Acid isolation kit (Ambion, Austin, TX, USA), according to the manufacturer’s instructions but specifically leaving out the first step of isolation (deparaffinization). ctDNA was isolated from the plasma samples using a NucleoSpin Plasma XS kit (Macherey-Nagel, Dueren, Germany) according to the manufacturer’s instructions.

**Figure 1:**
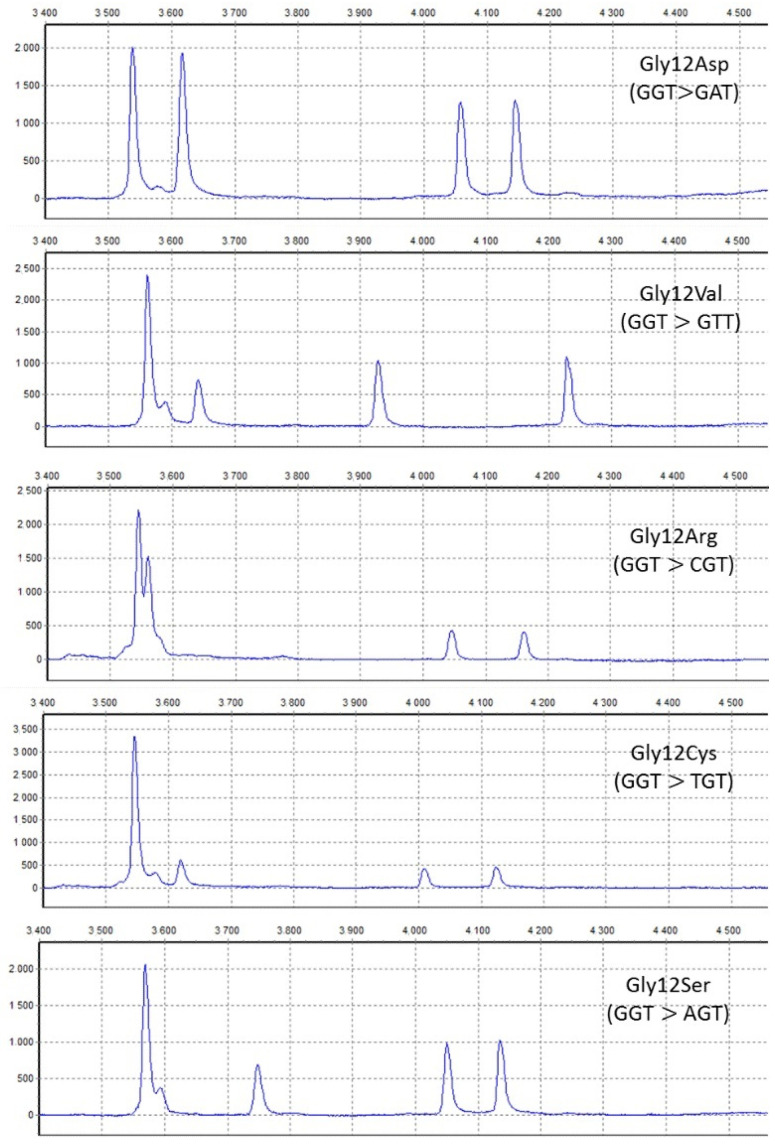
If a mutation is present in the sample (in this case *KRAS* mutation), four peaks are visible in the electropherogram: wildtype homoduplex, mutant homoduplex and two heteroduplexes (from left to right). The figure illustrates electropherograms of standard samples with the most commonly detected *KRAS* mutations in PDAC tissues. Note that different *KRAS* mutations have different positions of peaks in the electroferogram. If no mutation is present in the sample, only one peak corresponding to the wildtype homoduplex is visible in the electropherogram.

Somatic mutations in *KRAS* exons 12 and 13 were detected using the PCR method followed by the formation of heteroduplexes and subsequent heteroduplex analysis using capillary gel electrophoresis at an optimal temperature using the ABI Prism 3100 (Applied Biosystems, Waltham, MA, USA), as in previous studies [10,11,12]. Details can be found in Supplementary Materials and Methods. The heteroduplex analysis results were visualized using Gene Marker v2.4.2, and individual types of KRAS mutations were identified by comparing them to standard DNAs with specific mutant alleles (see Figure 1).

### 2.3. Statistical Analysis

The mean age of the patients was calculated using the mean and standard deviation. Survival curves were constructed using the Kaplan–Meier method and described by the median survival and its 95% CI for all patient groups tested. Comparison of survival curves was conducted using the log-rank test. A *p* value ˂ 0.05 was considered statistically significant. The software MedCalc v 19.1.7 was used for statistical analysis.

## 3. Results

Basic patient characteristics are summarized in Table 1.

*KRAS* mutations were detected in 90% (106/118) of tissue samples and in 44% (20/45) of plasma samples. All mutations were found in exon 2, codon 12 (see Table 2).

### 3.1. Tumor Tissue Samples

**Figure 2:**
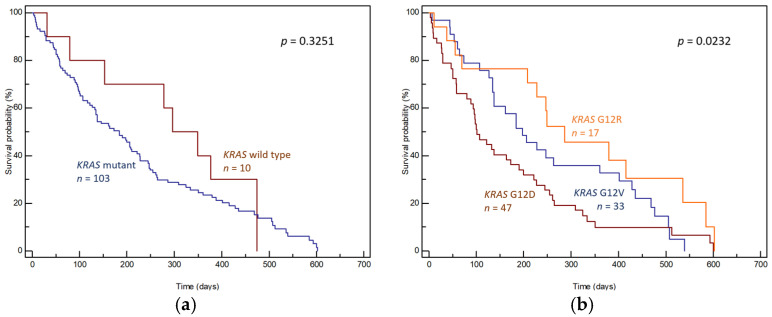
Overall survival curves (**a**) according to the presence of *KRAS* mutations in tumor tissue and (**b**) according to the presence of the three most common *KRAS* mutation subtypes in tumor tissue.

In patients with known survival time (*n* = 116), the OS of patients with detected *KRAS* mutations (*n* = 106) was compared to that of patients with no such mutations (*n* = 10). To ensure the homogeneity of the group, individuals with two *KRAS* mutations (*n* = 2, OS 155 and 186 days) and one patient who was additionally diagnosed with a second type of cancer (rectal cancer, OS 122 days) were excluded. Comparing the OS of patients with and without *KRAS* mutation in tumor tissue, no statistically significant difference was achieved (median OS 184 days, 95% CI: 31–474 vs. 296 days, 95% CI: 31–474, *p* = 0.3251, Figure 2a).

However, the overall survival of patients varied significantly depending on the specific type of *KRAS* mutation. The median OS times of patients with the three most common *KRAS* mutations, namely, G12D, G12V and G12R, were 101 days (95% CI: 80–600), 198 days (95% CI: 134–540) and 286 days (95% CI: 70–602), respectively, *p* = 0.0232 (Figure 2b).

**Figure 3:**
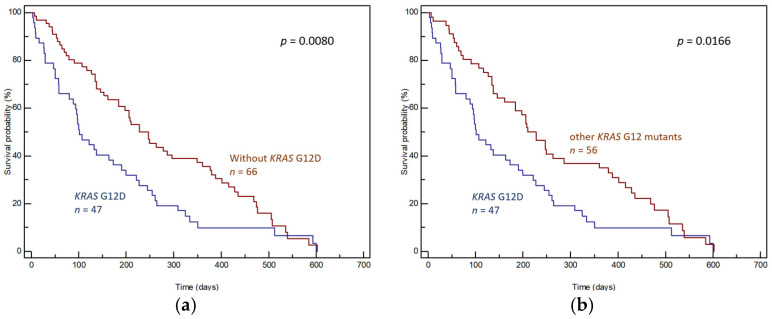
Differences in OS of (**a**) patients with and without *KRAS* G12D in tumor tissue and (**b**) patients with *KRAS* G12D and patients with other *KRAS* mutations in tumor tissue.

**Figure 4:**
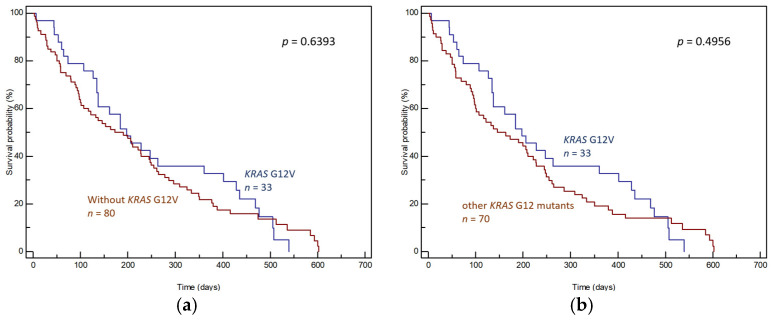
Differences in OS of (**a**) patients with and without *KRAS* G12V in tumor tissue and (**b**) patients with *KRAS* G12V and patients with other *KRAS* mutations in tumor tissue.

**Figure 5:**
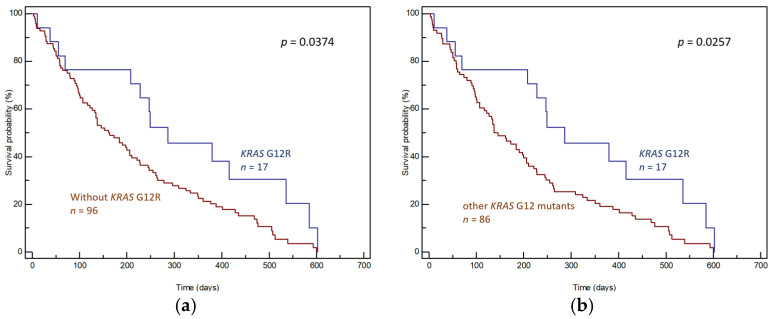
Differences in OS of (**a**) patients with and without *KRAS* G12R in tumor tissue and (**b**) patients with *KRAS* G12R and patients with other *KRAS* mutations in tumor tissue.

The effects of individual mutations on prognoses were determined by comparing the OS of patients with the given mutation and patients without the given mutation (wild-type *KRAS* + other *KRAS* mutations; Figure 3a, Figure 4a and Figure 5a) and by comparing the OS of patients with the given mutation and patients with other *KRAS* mutations (Figure 3b, Figure 4b and Figure 5b).

Regarding the effect of the individual *KRAS* mutations, the G12D mutation was found to correlate significantly with a worse prognosis. This was apparent in comparisons with both the group lacking *KRAS* G12D (median OS 101 days, 95% CI: 80–600 vs. 228 days, 95% CI 184–602, *p* = 0.0080, Figure 3a) and the group with other *KRAS* mutations (median OS 101 days, 95% CI: 80–600 vs. 210 days, 95% CI: 161–602, *p* = 0.0166, Figure 3b). In addition, the prognosis of patients harboring *KRAS* G12D was significantly worse than that of other participants regardless of whether patients underwent radical surgery (Supplementary Figure S1a, *p* = 0.0075) or not (Supplementary Figure S1b, *p* = 0.0113).

The G12V mutation did not correlate with prognosis, whether compared to all patients without this mutation (median OS 198 days, 95% CI: 134–540 vs. 173 days, 95% CI: 101–602, *p* = 0.6393, Figure 4a) or compared to patients with other *KRAS* mutations (median OS 198 days, 95% CI: 134–540 vs. 146 days, 95% CI: 98–602, *p* = 0.4956, Figure 4b).

In contrast, the G12R mutation correlated with better prognosis. Patients with G12R had significantly longer median OS than patients without G12R (median OS 286 days, 95% CI: 70–602 vs. 162 days, 95% CI: 122–600, *p* = 0.0374, Figure 5a) or patients with other *KRAS* mutations (median OS 286 days, 95% CI: 70–602 vs. 137 days, 95% CI: 107–600, *p* = 0.0257, Figure 5b). The positive effect of G12R on survival was also significant in patients treated with radical surgery (Supplementary Figure S2a, *p* = 0.0408); however, it was suppressed in the subset of patients who did not undergo surgery (Supplementary Figure S2b, *p* = 0.3090).

### 3.2. ctDNA Samples

**Figure 6:**
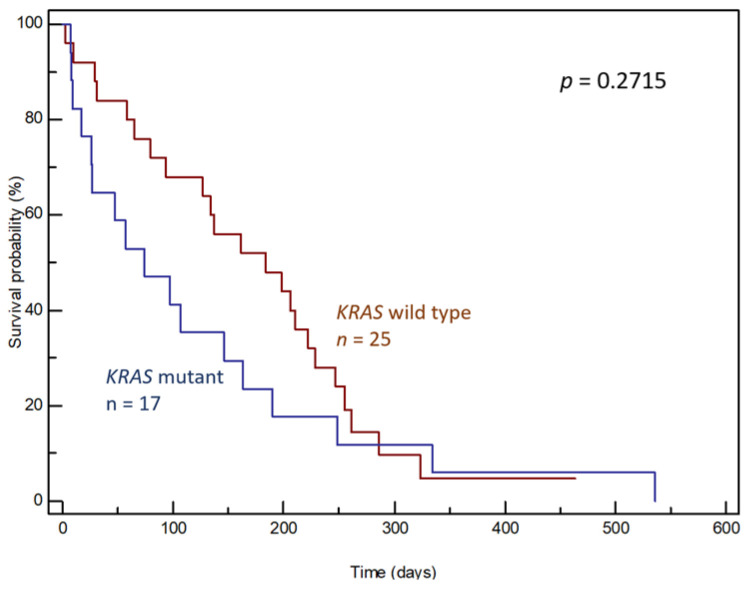
Overall survival curves according to the presence of *KRAS* mutations in ctDNA.

Patient survival was compared based on the presence or absence of *KRAS* mutations in plasma samples. In general, the presence of a *KRAS* mutation in ctDNA had no significant effect on patient survival (median OS 74 days, 95% CI: 17–536 vs. 184 days, 95% CI: 94–228, *p* = 0.2715, Figure 6).

**Figure 7:**
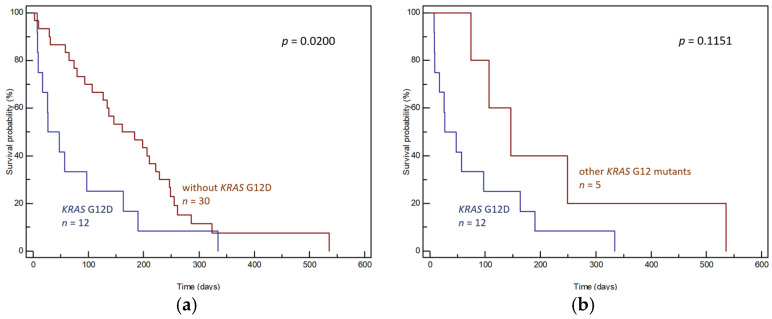
Differences in OS of (**a**) patients with and without *KRAS* G12D in ctDNA and (**b**) patients with *KRAS* G12D and patients with other *KRAS* mutations in ctDNA.

Additionally, the effect of the most commonly detected mutation, G12D, on OS was assessed. Patients with a detected G12D mutation had a significantly shorter median survival rate than other patients (median OS 27 days, 95% CI: 8–334 vs. 161 days, 95% CI: 107–536, *p* = 0.0200, Figure 7a), and a similar trend was evident when compared to patients with other *KRAS* mutations, although this trend lacked statistical significance (median OS 27 days, 95% CI: 8–334 vs. 146 days, 95% CI: 74–536, *p* = 0.1151, Figure 7b). No other analyses were performed, given the low number of ctDNA samples.

## 4. Discussion

*KRAS* mutations are found in various types of cancer, but their frequency is diverse. Most commonly among all malignancies, *KRAS* is mutated in PDAC tissue (66% according to the Catalogue of Somatic Mutations in Cancer, COSMIC [27], or 90% according to this study and similar recent studies, e.g., [4,28,29]); furthermore, *KRAS* mutations are common in colorectal tumors (33%) and lung cancer (16%) [27]. The distribution of specific types of *KRAS* mutations is similarly heterogeneous. In this study, in accordance with similar studies [15,16,21,22] and COSMIC [27], we showed that the three most common mutations found in PDAC tissue were G12D, G12V and G12R, unlike colorectal cancer, where the most common mutations are G12D, G12V and G12C, and lung cancer, where G12C is most common, followed by G12V and G12D [27]. Previously, this variability was the subject of several studies, gradually revealing the heterogeneous behavior of individual mutant Ras proteins. Some *KRAS* mutations cause tumors to behave more aggressively than others [30,31], and the various *KRAS* mutations exhibit different sensitivity levels to medications [32,33,34] or cause activation of different downstream signaling effectors [19].

The importance of individual mutant forms of *KRAS* has also recently come to light thanks to the tremendous success of pharmacological research. After decades of rather unsuccessful effort to find a treatment targeted against the Kras oncoprotein [35,36], an inhibitor of Kras G12C, which occurs in 13.6% of all *KRAS*-mutated tumors, was found [37]. This led to the clinical validation of two targeted therapies: sotorasib and adagrasib [38,39,40]. This is particularly good news for the treatment of lung cancer, where G12C is the most common *KRAS* subtype and accounts for about half of all *KRAS* mutation types [41,42]. In PDAC, unfortunately, the incidence of G12C is only about 1% [4,37] (5% in this study). However, the importance of specific *KRAS* mutations, rather than just the division into mutated and non-mutated, has become clear.

We have reported in the past that *KRAS* mutations detected in pancreatic masses themselves do not show any prognostic value [11], and we confirmed this finding again in this study with a new group of patients. We also show here that patients with *KRAS* G12D mutations have significantly shorter survival rates than patients without this mutation, irrespective of whether they have undergone surgical treatment. Similar results linking G12D to worse OS in a single-center study have been published by several authors. Bournet et al. [15] studied a group of patients with advanced unresectable PDAC tumors treated with chemotherapy or supportive care. In accordance with our study, the authors reported that the G12D *KRAS* mutation was an independent negative prognostic marker both in the entire patient group and in the subgroup of patients treated with chemotherapy. With respect to the above and to our results, the G12D mutation seems to be a therapy-independent negative prognostic factor. In their study, Bournet et al. also used EUS-FNB materials, although their materials took the form of fresh cellular aspirate remaining in the needle catheter after cytological and histological examination, while in our study we used cytological slides, which are more appropriate for determining *KRAS* mutations based on our experience, particularly given their low rate of false negatives [12]. This may explain the higher rate of *KRAS* mutations captured in our study vs. the study of Bournet et al. (90% vs. 67%). Similar results were also reported by Oldani et al. [14], who used the samples of resected PDAC tumors of elderly patients undergoing radical surgical procedures. The authors observed a trend of a shorter survival rates in patients with the G12D *KRAS* mutation, although statistical significance was not reached. Other examples of studies reporting significantly worse OS and disease-free survival (DFS) in patients with *KRAS* G12D are a recent study by Dai et al. [21] and one by McIntyre et al. [43] conducted on PDAC surgical specimens collected during radical surgery, as well as a current large-sample-size study by Yousef et al. [22]. In Yousef’s study, samples from 803 patients in different stages of PDAC were retrospectively analyzed, and *KRAS* G12D mutant tumors were associated with significantly worse OS. On the other hand, another recent study reported that the *KRAS* G12D mutation subtype was not significantly associated with worse survival in PDAC patients globally; only patients with resectable disease harboring G12D had shorter median survival than other PDAC patients in the aggregate [44].

Regarding the *KRAS* G12R mutation, this study revealed significantly improved OS survival in PDAC patients with this mutation. Its positive prognostic effect was reflected in the group of all patients as well as in the subgroup of operated patients, but surprisingly, it was not shown in the group of non-operated patients. It is therefore possible that the improved OS of the *KRAS* G12R was enhanced by radical surgery. However, this hypothesis should be tested on a larger group of patients. Similar to our results, improved OS and progression free survival (PFS) for *KRAS* G12R were also reported by Diehl et al. in a group of patients with locally advanced or metastatic PDAC [45]. Another study supporting our findings is the abovementioned study by Yousef et al., where patients with *KRAS* G12R and wild type *KRAS* had improved OS compared to patients with *KRAS* G12D [22]. An opposite effect of *KRAS* G12R was seen in an earlier study performed in the Japanese population, where the G12D and G12R mutations were negative prognostic factors for OS [46].

In the same manner as the authors of several recent studies [17,47,48,49,50,51,52,53,54,55], we applied the concept of determining the prognosis of patients with PDAC according to the *KRAS* mutation type found in ctDNA. For this purpose, we used plasma samples from patients with metastatic PDAC, who usually have the worst performance status and are therefore most burdened by traditional biopsy. The overall rate of captured mutations was lower with ctDNA than with EUS-FNB (44% vs. 90%), which is in agreement with the results of similar studies, where it ranges between 26% [47] and 72.3% [51]. The results of the analyses were similar to those using EUS-FNB samples, and the presence of *KRAS* mutation in plasma had no considerable impact on the overall survival of the patients; however, patients with the G12D subtype had a statistically significantly shorter OS than the other patients. Circulating tumor DNA can thus be used as an alternative to EUS-FNB as a source from which to determine the patient prognosis based on various *KRAS* mutations.

Similar studies determining *KRAS* mutations in the plasma of patients with PDAC have been conducted predominantly in the Asian population [17,48,49,50,51,52,54,55], and their results vary. Some authors did not observe any significant association between the presence of a *KRAS* mutation and survival in agreement with our findings [49,51], but some of them observed worse OS in patients with a detected *KRAS* mutation in ctDNA [52,53,54,55], while others did not observe any effect of *KRAS* mutations on survival based on tissue sample analysis but did observe significantly shorter survival in patients with a *KRAS* mutation based on plasma assessment [17,48,50]. Thus far, only one pilot study has been conducted in a European population [47]; it reports a significantly shorter OS for patients with *KRAS* mutations in plasma but does not focus on individual *KRAS* mutations. Regarding individual mutations, Kinugasa et al. [48] and Cheng et al. [51] observed significantly shorter survival in patients with G12V, but Hadano et al. [50] reported no differences in OS by *KRAS* subtype, whereas Guo et al. [55] observed significantly shorter OS in *KRAS* G12D patients, in agreement with our study. To our knowledge, this is the first study focused on the determination of any prognostic roles of individual *KRAS* mutation types in plasma samples in the European population.

We are aware of the limitations in our study, resulting mainly from the smaller cohort of patients and thus its division into even smaller subgroups having individual *KRAS* mutations. The results may, therefore, not have sufficient statistical power and should not be generalized. However, this is not unusual, as shown by similar works studying *KRAS* mutations in PDAC patients and often reaching barely 70 tissue samples or 50 ctDNA samples [14,18]. The present study tried to maintain, as much as possible, a rational testing procedure that could feasibly be implemented in clinical practice. We worked with a set of real patients, eliminating only patients with no known survival time and patients with severe comorbidity (colorectal cancer). We utilized FNB samples that are routinely available from PDAC diagnostics and verified the potential prognostic role of a single genetic marker, *KRAS*, which is routinely determined in all molecular biological and diagnostic laboratories. Lack of information about the presence of other somatic mutations can be seen as another limiting factor. However, wide genomic analysis of the samples was not the aim of the study and would not even be implementable in clinical practice, given the increased requirements for sample quantity and quality and the need for instrumentation that is not commonly found in routine diagnostic laboratories. Moreover, with regard to real clinical practice, blood samples were only collected from patients with stage IV disease, in which the potential investigation of liquid biopsy instead of tissue biopsy makes the most sense. Therefore, the number of samples for ctDNA analysis was also relatively low.

## 5. Conclusions

The prognosis of PDAC patients appears to be dependent on the specific subtype of *KRAS* mutation present in the tumor. In our cohort of 118 patients, G12D (GGT > GAT) confers the worst prognosis and G12R (GGT > CGT) the best prognosis, resulting in a shortening and prolongation of median survival by approximately half (4 months) in patients with the G12D and the G12R mutations, respectively. *KRAS* testing from EUS-FNB cytology slides, or from plasma samples in cases where tissue biopsy is contraindicated, could represent a useful tool in the rational decision-making process of PDAC therapy.

## Figures and Tables

**Table 1:** Characteristics of studied group of patients.

Monitored Characteristic		Number of Patients
All cases		118 (100%)
Age		67.9 ± 9.4
Gender	Male	64 (54%)
	Aged	44–92 years, median 69
	Female	54 (46%)
	Aged	44–87 years, median 66
Location of PDAC	Head	81 (69%)
	Body	28 (24%)
	Tail	9 (8%)
Disease stage	II	3 (3%)
	III	56 (47%)
	IV	58 (49%)
	Unknown	1 (1%)
Treatment	Surgery + adjuvant chemotherapy	16 (14%)
	Surgery alone	11 (9%)
	Chemotherapy alone	44 (37%)
	Supportive care	44 (37%)
	Unknown	3 (3%)

**Table 2:** *KRAS* mutation tumor tissue and plasma samples.

Result of *KRAS* Analysis	Tissue Sample (*n* = 118)	Plasma Sample (*n* = 45)
*KRAS* wild type	12/118 (10%)	25/45 (56%)
*KRAS* mutant	106/118 (90%)	20/45 (44%)
GGT/GAT (G12D)	47/106 (44%)	13/20 (65%)
GGT/GTT (G12V)	33/106 (31%)	2/20 (10%)
GGT/CGT (G12R)	18/106 (17%)	2/20 (10%)
GGT/TGT (G12C)	5/106 (5%)	0
GGT/AGT (G12S)	1/106 (1%)	1/20 (5%)
GGT/GTT (G12V) + GGT/AGT (G12S)	1/106 (1%)	1/20 (5%)
GGT/GAT (G12D) + GGT/AGT (G12S)	1/106 (1%)	1/20 (5%)

## Data Availability

Technical and clinical data presented in this study are available from the corresponding author at lbenesova@genomac.cz.

## Supplementary Materials

The following supporting information can be downloaded at: https://www.mdpi.com/article/10.3390/genes15101302/s1, Supplementary Materials and Methods, Figure S1: Differences in OS of patients with and without *KRAS* Gly12Asp in tumor tissue (a) in the subgroup of operated patients and (b) in the subgroup of non-operated patients. Figure S2: Differences in OS of patients with and without *KRAS* Gly12Arg in tumor tissue (a) in the subgroup of operated patients and (b) in the subgroup of non-operated patients.
