# Supplementary material for: Prognostic Role of Specific KRAS Mutations Detected in Aspiration and Liquid Biopsies from Patients with Pancreatic Cancer
Source: Genes (Basel). 2024 Oct 7;15(10):1302. doi: 10.3390/genes15101302 (PMC11507146; doi:10.3390/genes15101302)
Supplement: Supplementary file 1 [file genes-15-01302-s001.zip › genes-3192336-supplementary.pdf]

Supplementary Materials and Methods:

The PCR reaction mixture consisted of 5 µl of 2x PPP master mix (Top-Bio, Prague, CZ), 2 µl of water, 1 µl of template DNA and 1 µl of each primer (5 µM). The left primer was fluorescently labeled and the right primer contained 40bp long GC-clamp (left primer: 5′- FL-ATGACTGAATATAAACTTGTG-3′, right primer: 5′- [GC]-CCTCTATTGTTGGATCATATTC-3′). The PCR product was subsequently denatured and slowly reassociated to form homoduplexes and heteroduplexes. Heteroduplex analysis was performed under the following separation conditions: sample injection 2kV, 10s; analysis of sample 15kV, 30min at 50 ° C.

Supplementary Figures:

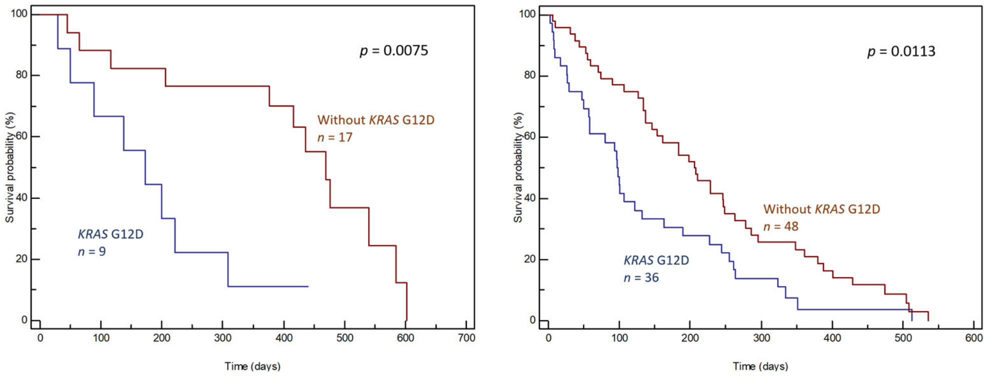

(a) (b)

Figure S1. Differences in OS of patients **(a)** with and without *KRAS* Gly12Asp in tumor tissue in the subgroup of operated patients **(b)** with and without *KRAS* Gly12Asp in tumor tissue in the subgroup of non-operated patients.

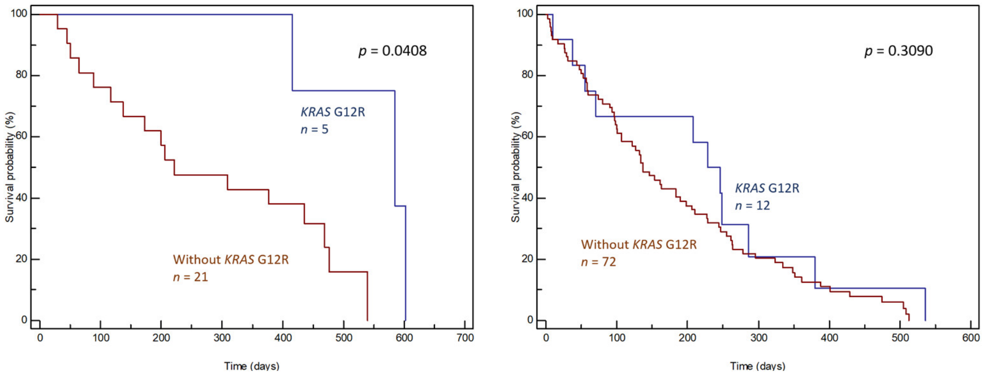

(a) (b)

Figure S2. Differences in OS of patients **(a)** with and without *KRAS* Gly12Arg in tumor tissue in the subgroup of operated patients **(b)** with and without *KRAS* Gly12Arg in tumor tissue in the subgroup of non-operated patients.
